# Integrating behavioural thermoregulatory strategy into the animal personality framework using the common lizard, *Zootoca vivipara* as a model

**DOI:** 10.1038/s41598-024-64305-z

**Published:** 2024-06-20

**Authors:** Gergely Horváth, Tibor Sos, Gábor Bóné, Csanád Endre Lőrincz, Péter László Pap, Gábor Herczeg

**Affiliations:** 1 Department of Systematic Zoology and Ecology, Institute of Biology, ELTE Eötvös Loránd University, Pázmány Péter sétány 1/c, 1117 Budapest, Hungary; 2 HUN-REN–ELTE–MTM Integrative Ecology Research Group, Pázmány Péter sétány 1/C, 1117 Budapest, Hungary; 3 Evolutionary Ecology Group, Hungarian Department of Biology and Ecology, Centre for Systems Biology, Biodiversity and Bioresources, Babeş-Bolyai University, Clinicilor street 5-7, Cluj-Napoca, Romania; 4 “Milvus Group” Bird and Nature Protection Association, B-dul 1 Decembrie 1918 121, 540445 Tîrgu Mureș, Romania; 5 Department of Physiology, Anatomy and Neuroscience, Faculty of Science and Informatics, University of Szeged, Közép fasor 52, 6726 Szeged, Hungary

**Keywords:** Animal personality, Behavioural consistency, Thermoregulatory strategy, Behavioural syndrome, Behavioural predictability, Evolution, Behavioural ecology, Herpetology

## Abstract

The study of consistent between-individual behavioural variation in single (animal personality) and across two or more behavioural traits (behavioural syndrome) is a central topic of behavioural ecology. Besides behavioural type (individual mean behaviour), behavioural predictability (environment-independent within-individual behavioural variation) is now also seen as an important component of individual behavioural strategy. Research focus is still on the ‘Big Five’ traits (activity, exploration, risk-taking, sociability and aggression), but another prime candidate to integrate to the personality framework is behavioural thermoregulation in small-bodied poikilotherms. Here, we found animal personality in thermoregulatory strategy (selected body temperature, voluntary thermal maximum, setpoint range) and ‘classic’ behavioural traits (activity, sheltering, risk-taking) in common lizards (*Zootoca vivipara*). Individual state did not explain the between-individual variation. There was a positive behavioural type—behavioural predictability correlation in selected body temperature. Besides an activity—risk-taking syndrome, we also found a risk-taking—selected body temperature syndrome. Our results suggest that animal personality and behavioural syndrome are present in common lizards, both including thermoregulatory and ‘classic’ behavioural traits, and selecting high body temperature with high predictability is part of the risk-prone behavioural strategy. We propose that thermoregulatory behaviour should be considered with equal weight to the ‘classic’ traits in animal personality studies of poikilotherms employing active behavioural thermoregulation.

## Introduction

By directly affecting biochemical processes, body temperature influences metabolic and developmental rates^1–3^, as well as reproductive success^4,5^. Consequently, it also affects distribution and abundance patterns in ecosystems^6,7^. As the body temperature of small-bodied poikilotherms (i.e., animals whose internal temperature varies considerably), comes to equilibrium with that of their environment rapidly, the quality of thermal (micro)habitats and thermoregulatory strategy have particular importance for these taxa^8,9^. To cope with environmental temperature fluctuations and maintain optimal body temperatures, poikilotherms rely on physiological and behavioural adaptations, the latter being dominant for small-bodied vertebrate taxa^8,10–16^. Thus, thermoregulatory behaviour is an essential aspect of poikilotherms’ life, which can have cascading effects on predator–prey interactions, as well as on competition among species, ultimately shaping community dynamics^17^. Even though the benefits of behavioural thermoregulation (optimal performance) are self-explanatory, it also has inherent costs (lost opportunities in foraging and mating; exposure to predators)^8,12,13^.

Consistent between-individual behavioural differences across various ecological situations and over time in single (animal personality) or across more functionally different behaviours (behavioural syndrome) has been a hot topic of evolutionary behavioural ecology in the last two decades (for recent reviews and meta-analyses see^18–21^). While between-individual behavioural variation is traditionally approached by focusing on *behavioural type* (e.g., how aggressive an individual is on average), considerable research effort has been made to reveal the eco-evolutionary relevance of the within-individual behavioural variation too. As a result, *behavioural predictability* (e.g., how much an individual’s aggression varies irrespective of the environment) is now recognized as a valid and important component of an individual’s behavioural strategy^22–26^. Animal personality researchers often rely on the ‘Big Five’ framework when studying behavioural consistency, targeting five main behavioural traits: movement activity, exploration, risk-taking, sociability and aggression, sensu Réale et al.^27^. However, there are other fitness-linked behaviours to consider, including thermoregulatory behaviour in poikilotherms, which arguably have high importance for these taxa. Further, considering the number of potential trade-offs between thermoregulation and other fitness-linked behavioural, physiological and life-history traits^8,11,28–30^, the emergence of individual strategies is expected.

Consequently, there is a growing interest in the integration of thermoregulatory behaviour (and thermal physiology) into animal personality studies, as it provides valuable insights into the individual variation in behavioural responses to environmental challenges^31–35^. Previous experimental data show that there is considerable inter-individual variation in voluntarily selected body temperature (the “goal” of behavioural thermoregulation in reptiles^9,32,36–39^), indicating the presence of between-individual variation in thermal strategies. This variation can be driven by various mechanisms. For instance, Sih et al.^40^ suggested that individual variation in inherently stable and/or labile traits that are linked to individual state (e.g., sex, age, physiological state) might play important role in the emergence of behavioural consistencies. To date, studies testing thermoregulatory strategies in the context of behavioural syndromes are rather scarce (see also^41^). Goulet et al.^32,42^ and Michelangeli et al.^35^ proposed that organisms can be aligned along a ‘hot–cold’ axis: they showed that delicate skinks (*Lampropholis delicata*) with a ‘hot’ thermal type are bolder, having higher maximal sprint speed, perform better at higher optimal temperatures and within a narrower range compared to ‘cold’ type peers. Bolder individuals preferring higher temperatures seem to be rather general across poikilotherms (reptiles:^34,36,43,44^; and fishes^45,46^), nevertheless, some studies are indicating the opposing pattern^33,47^. It is straightforward to assume a metabolic link in the background of thermal behavioural types^32,35,42^. Nevertheless, available studies considering metabolic rate show a controversial picture, as there was seemingly no link between metabolic rate and preferred temperature in common lizards *Zootoca vivipara*^37^, while in larvae of *Ischnura elegans* damselfly individuals with faster metabolic rate and growth had lower thermal optima for metabolic performance^48^. These pioneering studies are very promising, and there is a need for more studies where poikilotherm behavioural thermoregulation is studied in the animal personality framework together with ‘classic’ behavioural traits to better understand whether components of thermoregulatory behaviour are consistent individual traits and if so, whether thermoregulatory and ‘classic’ behavioural traits form a syndrome.

Reptiles are prime models for studying thermoregulatory personality, because reptile thermal ecology and physiology are well studied, particularly in small-bodied, heliothermic lizards that maintain their body temperature mainly by timing their activity, microhabitat use, and posture, rather than by physiological adjustments^8,10,11,15,49^. However, studies that investigate thermoregulatory behaviour in the context of animal personality are still scarce. A potential problem is that relatively low number of repeats per individual (˂ 6) is collected typically, which makes the estimation of behavioural type and predictability in thermoregulatory behaviour statistically difficult, while in those studies where sample sizes is suitable for such statistical methods^50–52^, behavioural predictability is not examined. Hence, there’s virtually no information on the within-individual component of thermoregulatory strategy variation. Links between behavioural type and behavioural predictability in behavioural traits are commonplace in nature, showing variability in both strength and direction^24,26^, while there is no such information available regarding thermoregulatory behaviour.

Here, we aim to reveal the associations between behavioural thermoregulatory strategy (i.e., thermal preference and thermoregulatory precision) and ‘classic’ personality traits (movement activity, sheltering and risk-taking) in a small-bodied poikilotherm, testing behavioural types and predictability. Our model species was the common lizard (*Zootoca vivipara*), a viviparous small-bodied (50–70 mm adult SVL) lacertid, native to most of Northern Eurasia and being among the most studied reptiles^53–60^. *Zootoca vivipara* is an excellent model for our purposes, as it successfully occupies low-thermal quality (high altitude and latitude) habitats and is known as one of the most effective thermoregulators^12,13,61^.

Thermoregulatory behaviour was described using three variables: selected body temperatures (T_sel_; the ‘goal’ of thermoregulation), voluntary thermal maximum (T_Vmax_; a proxy for thermal tolerance) and (iii) the width of the set-point range (T_set_; a measure of thermoregulatory precision). Activity was described using two variables: sheltering (time spent in shelter) and movement activity (movements when not being in shelter). Risk-taking was estimated by the time needed to leave the shelter after a simulated attack.

We were interested in whether (1) there are consistent between-individual differences (animal personality) present in components of thermoregulatory strategy and ‘classic’ behavioural traits of this species. Further, as available information suggests that thermoregulatory strategy depends heavily on individual state in reptiles, for instance, individuals with intense blood-parasite burden (like *Karyolysus* and *Schellackia*) were shown to be imprecise thermoregulators^34,62^, we tested whether (2) various traits linked to individual state (i.e., body size, relative head size, condition and blood parasite infection intensity) affect behavioural type and behavioural predictability. Additionally, we tested whether (3) there are correlations between behavioural type and predictability of traits describing thermoregulatory strategy and behaviour. Lastly, we were interested whether (4) there are between-individual correlations (behavioural syndromes) within and between thermoregulatory and ‘classic’ behavioural traits. Owing to the complexity of our questions, we do not form explicit hypotheses and predictions regarding all potential relationships, but we hypothesized consistent between-individual variation being present not only in ‘classic’ behavioural traits, but also in thermoregulatory strategy (i.e., animal personality can be found in thermoregulation). We also hypothesized that thermoregulatory and behavioural strategies are integrated (i.e., general behaviour and thermoregulation form a syndrome). Specifically, based on the previously proposed ‘hot–cold’ axis, we predicted a positive correlation between risk-taking and thermal preference.

## Results

### Animal personality

We found that individuals substantially differed in all three traits describing thermoregulatory strategy and in the studied ‘classic’ behavioural traits as well (sd_Intercept_; see Table 1 and Electronic Supplementary Material Fig. S1). The population level average T_sel_ was estimated to be 32.22 °C (back-transformed intercept for T_sel_ *β*_*0*_ in Table 2; ranging between 29.69 and 33.5 °C), while average T_set_ was 4.49 °C (range: 3.33 to 6.29 °C) and average T_Vmax_ was 39.73 °C (range: 38.38 to 40.82 °C). Regarding ‘classic’ behavioural traits, average movement activity (i.e., number of transitions between squares in the test arena) was 6.84 (range: 3.87 to 10.58), average sheltering was 75.40 s (range: 2.62 to 432.75) and average risk-taking was 195.58 s (range: 56.29 to 677.26). Repeatability estimates from separate DHMMs indicate *Z. vivipara* being highly (based on meta-analytic results from Bell et al.^63^) consistent in T_sel_ (mean repeatability [95% credible interval] = 0.66 [0.42 to 0.9]), as well as in T_set_ and T_Vmax_ (0.83 [0.63 to 0.99] and 0.87 [0.56 to 1.0], respectively). In the case of ‘classic’ behavioural traits, estimates indicate high consistency in both activity (0.64 [0.39 to 0.87]), sheltering (0.69 [0.49 to 0.92]) and risk-taking (0.82 [0.59 to 0.99]).

**Table 1:** Estimates and 95% credible intervals (in parentheses) of fixed and random effects (mean model) and residual standard deviation (residual model) of T_sel_, T_set_, T_Vmax,_ activity, sheltering and risk-taking in adult male *Zootoca vivipara*.

	T_sel_	T_set_	T_Vmax_	Activity	Sheltering	Risk-taking
Mean model						
Fixed effects						
Intercept	0.04 [− 0.26 to 0.33]	− 0.05 [− 0.29 to 0.21]	− 0.04 [− 0.25 to 0.16]	0.09 [− 0.25 to 0.47]	− 0.18 [− 0.53 to 0.16]	− 0.12 [− 0.4 to 0.15]
Time	**− 0.16 [− 0.3 to − 0.02]**	**0.25 [0.12 to 0.39]**	0.14 [− 0.02 to 0.29]	**− 0.12 [− 0.22 to − 0.005]**	− 0.06 [− 0.19 to 0.07]	**− 0.24 [− 0.41 to − 0.08]**
SVL	− 1.38 [− 4.21 to 1.43]	− 0.77 [− 3.13 to 1.52]	− 0.83 [− 2.69 to 1.04]	0.76 [− 2.48 to 3.99]	− 0.39 [− 3.32 to 2.66]	− 0.53 [− 3.09 to 2.17]
Relative head size	1.79 [− 1.26 to 4.93]	0.73 [− 1.94 to 3.44]	0.57 [− 1.67 to 2.78]	− 0.08 [− 3.89 to 3.71]	− 0.21 [− 3.73 to 3.24]	0.64 [− 2.45 to 3.63]
Condition	− 0.48 [− 1.92 to 0.91]	0.24 [− 0.89 to 1.37]	0.36 [− 0.56 to 1.32]	− 0.97 [− 2.54 to 0.59]	0.79 [− 0.67 to 2.19]	− 0.032 [− 1.34 to 1.27]
Parasite	0.04 [− 0.24 to 0.33]	− 0.04 [− 0.28 to 0.21]	0.02 [− 0.19 to 0.23]	0.002 [− 0.35 to 0.36]	− 0.22 [− 0.57 to 0.13]	0.02 [− 0.26 to 0.29]
SVL × Parasite	1.94 [− 3.28 to 6.88]	− 0.68 [− 4.92 to 3.48]	0.34 [− 3.19 to 3.89]	1.49 [− 4.29 to 7.39]	− 0.49 [− 5.72 to 4.77]	− 0.031 [− 4.36 to 4.31]
Relative head size × Parasite	− 2.49 [− 8.13 to 3.42]	0.52 [− 4.28 to 5.35]	− 0.98 [− 4.89 to 3.09]	− 2.49 [− 9.23 to 4.15]	1.45 [− 4.58 to 7.35]	1.26 [− 3.66 to 6.17]
Condition × Parasite	0.29 [− 1.05 to 1.69]	0.84 [− 0.54 to 2.26]	1.19 [0.12 to 2.26]	− 0.33 [− 1.88 to 1.19]	0.41 [− 1.08 to 1.83]	− 0.86 [− 2.23 to 0.46]
Random effects						
sd_Intercept_	**0.68 [0.42 to 1.01]**	**0.54 [0.31 to 0.82]**	**0.33 [0.06 to 0.61]**	**0.84 [0.6 to 1.19]**	**0.79 [0.54 to 1.11]**	**0.58 [0.34 to 0.86]**
sd_Time_	**0.23 [0.07 to 0.41]**	**0.16 [0.021 to 0.34]**	**0.18 [0.01 to 0.4]**	**0.08 [0.003 to 0.23]**	**0.14 [0.01 to 0.32]**	**0.13 [0.007 to 0.34]**
r_Intercept−Slope_	− 0.19 [− 0.86 to 0.63]	0.35 [− 0.59 to 0.93]	0.01 [− 0.83 to 0.82]	− 0.09 [− 0.89 to 0.82]	0.32 [− 0.64 to 0.91]	0.19 [− 0.77 to 0.91]
Residual model						
Fixed effects						
Intercept	**− 0.48 [− 0.71 to − 0.25]**	**− 0.23 [− 0.38 to − 0.08]**	− 0.09 [− 0.24 to 0.05]	**− 0.63 [− 0.92 to − 0.36]**	**− 0.52 [− 0.77 to − 0.29]**	**− 0.25 [− 0.47 to − 0.05]**
SVL	− 0.15 [− 2.25 to 1.96]	0.3 [− 1.29 to 1.84]	0.71 [− 0.86 to 2.17]	0.61 [− 1.91 to 3.23]	1.11 [− 1.28 to 3.39]	− 0.66 [− 2.43 to 1.00]
Relative head size	0.13 [− 2.19 to 2.42]	− 0.5 [− 2.25 to 1.32]	− 0.49 [− 2.25 to 1.34]	− 0.48 [− 3.39 to 2.59]	− 0.15 [− 2.77 to 2.48]	0.82 [− 1.19 to 2.98]
Condition	0.0503 [− 1.08 to 1.17]	0.28 [− 0.44 to 1.02]	− 0.18 [− 0.86 to 0.46]	− 0.28 [− 1.59 to 0.96]	**− 1.13 [− 2.17 to − 0.12]**	− 0.17 [− 1.11 to 0.78]
Parasite	0.0502 [− 0.19 to 0.29]	− 0.02 [− 0.17 to 0.15]	− 0.03 [− 0.18 to 0.13]	0.13 [− 0.14 to 0.42]	0.15 [− 0.08 to 0.38]	0.08 [− 0.13 to 0.28]
SVL × Parasite	− 1.53 [− 5.28 to 2.39]	1.23 [− 1.76 to 4.21]	1.65 [− 0.65 to 4.08]	− 0.34 [− 4.91 to 4.47]	0.79 [− 3.21 to 4.96]	− 1.81 [− 5.78 to 2.15]
Relative head size × Parasite	1.97 [− 2.47 to 6.15]	− 1.64 [− 5.01 to 1.83]	− 2.00 [− 4.79 to 0.61]	0.77 [− 4.72 to 5.99]	− 0.95 [− 5.62 to 3.69]	1.06 [− 3.31 to 5.46]
Condition × Parasite	− 0.11 [− 1.11 to 0.96]	0.48 [− 0.23 to 1.21]	0.15 [− 0.52 to 0.87]	− 0.72 [− 2.02 to 0.67]	− 0.81 [− 1.91 to 0.34]	0.82 [− 0.04 to 1.75]
Random effects						
ω^2^_Intercept_	**0.43 [0.22 to 0.69]**	**0.16 [0.01 to 0.37]**	**0.12 [0.005 to 0.31]**	**0.59 [0.34 to 0.93]**	**0.43 [0.24 to 0.68]**	**0.31 [0.04 to 0.59]**
r_Intercept–ω.Intercept_	**− 0.59 [− 0.92 to − 0.09]**	0.36 [− 0.61 to 0.93]	0.26 [− 0.76 to 0.94]	0.21 [− 0.35 to 0.69]	− 0.25 [− 0.78 to 0.41]	− 0.49 [− 0.94 to 0.27]
r_Slope–ω.Intercept_	0.15 [− 0.67 to 0.87]	0.14 [− 0.8 to 0.91]	− 0.09 [− 0.91 to 0.85]	0.08 [− 0.81 to 0.87]	− 0.006 [− 0.85 to 0.83]	− 0.17 [− 0.91 to 0.82]

Estimates are based on double hierarchical mixed models. Estimates which were different from 0 based on their 95% credible intervals are bolded.

**Table 2:** Between- and within-individual correlation among traits describing thermoregulatory behaviour, activity, sheltering and risk-taking in adult male *Zootoca vivipara*.

	Between-individual	Within-individual
T_sel_–T_set_	r = − 0.27	r = 0.03
	CI = − 0.59 to 0.14	CI = − 0.14 to 0.13
T_sel_–T_Vmax_	**r = 0.45** **CI = 0.07 to 0.78**	r = 0.09 CI = − 0.02 to 0.24
T_set_–T_Vmax_	r = − 0.04	r = 0.35
	CI = − 0.24 to 0.56	CI = 0.24 to 0.47
T_sel_–Activity	r = 0.33	r = − 0.03
	CI = − 0.02 to 0.61	CI = − 0.17 to 0.1
T_sel_–Sheltering	r = − 0.37	r = − 0.05
	CI = − 0.63 to 0.03	CI = − 0.19 to 0.13
T_sel_–Risk-taking	**r = − 0.34**	r = 0.01
	**CI = − 0.71 to − 0.02**	CI = − 0.13 to 0.19
T_set_–Activity	r = − 0.33	r = 0.09
	CI = − 0.52 to 0.19	CI = − 0.14 to 0.2
T_set_–Sheltering	r = 0.15	r = 0.04
	CI = − 0.2 to 0.45	CI = − 0.09 to 0.23
T_set_–Risk-taking	r = 0.09	r = − 0.004
	CI = − 0.34 to 0.47	CI = − 0.16 to 0.13
T_Vmax_–Activity	r = 0.14	r = -0.01
	CI = − 0.33 to 0.48	CI = − 0.19 to 0.15
T_Vmax_–Sheltering	r = − 0.14	r = − 0.12
	CI = − 0.56 to 0.29	CI = − 0.23 to 0.13
T_Vmax_–Risk-taking	r = − 0.22	r = 0.11
	CI = − 0.65 to 0.11	CI = − 0.06 to 0.28
Activity–Sheltering	**r = **− **0.77**	r = − 0.09
	**CI = **− **0.85 to **− **0.45**	CI = − 0.24 to 0.06
Activity–Risk-taking	**r = **− **0.59**	r = 0.02
	**CI = **− **0.76 to **− **0.25**	CI = − 0.14 to 0.15
Sheltering–Risk-taking	**r = 0.64**	r = 0.008
	**CI = 0.29 to 0.81**	CI = − 0.09 to 0.26

Significant between-individual correlations (indicators of a true behavioural syndrome) are in bold font. correlation coefficients and 95% credible intervals are shown.

### Effects of state variables

In general, behavioural types were not affected by any of the studied state-linked traits. We found indication of behavioural habituation (plasticity detected over time at the group level) only in the case of T_sel_ (− 0.16 [− 0.3 to − 0.02]), T_set_ (0.25 [0.12 to 0.39]), activity (− 0.12 [− 0.22 to − 0.005]) and risk-taking (− 0.24 [− 0.41 to − 0.08]): individuals selected lower body temperatures, but also expanding their set-point range by time, further, they decreased their activity and took more risk by time, i.e., they expressed habituation (data not shown). We found that for each trait, individuals substantially differ in how they change their behaviour over time (i.e., individual differences in habituation; sd_Time_; see Table 2), nevertheless, this change was independent of individual behavioural type (r_Intercept–Slope_; see Table 2) or behavioural predictability (r_Slope–ω.Intercept_; Table 2). We found a negative correlation between sheltering predictability and body condition − 1.13 [− 2.17 to − 0.12]): low-condition individuals are less predictable than their high-condition peers. None of the other state-linked traits affected rIIV of any studied behavioural trait (see Table 1).

### Correlations between behavioural type and predictability

**Figure 1:**
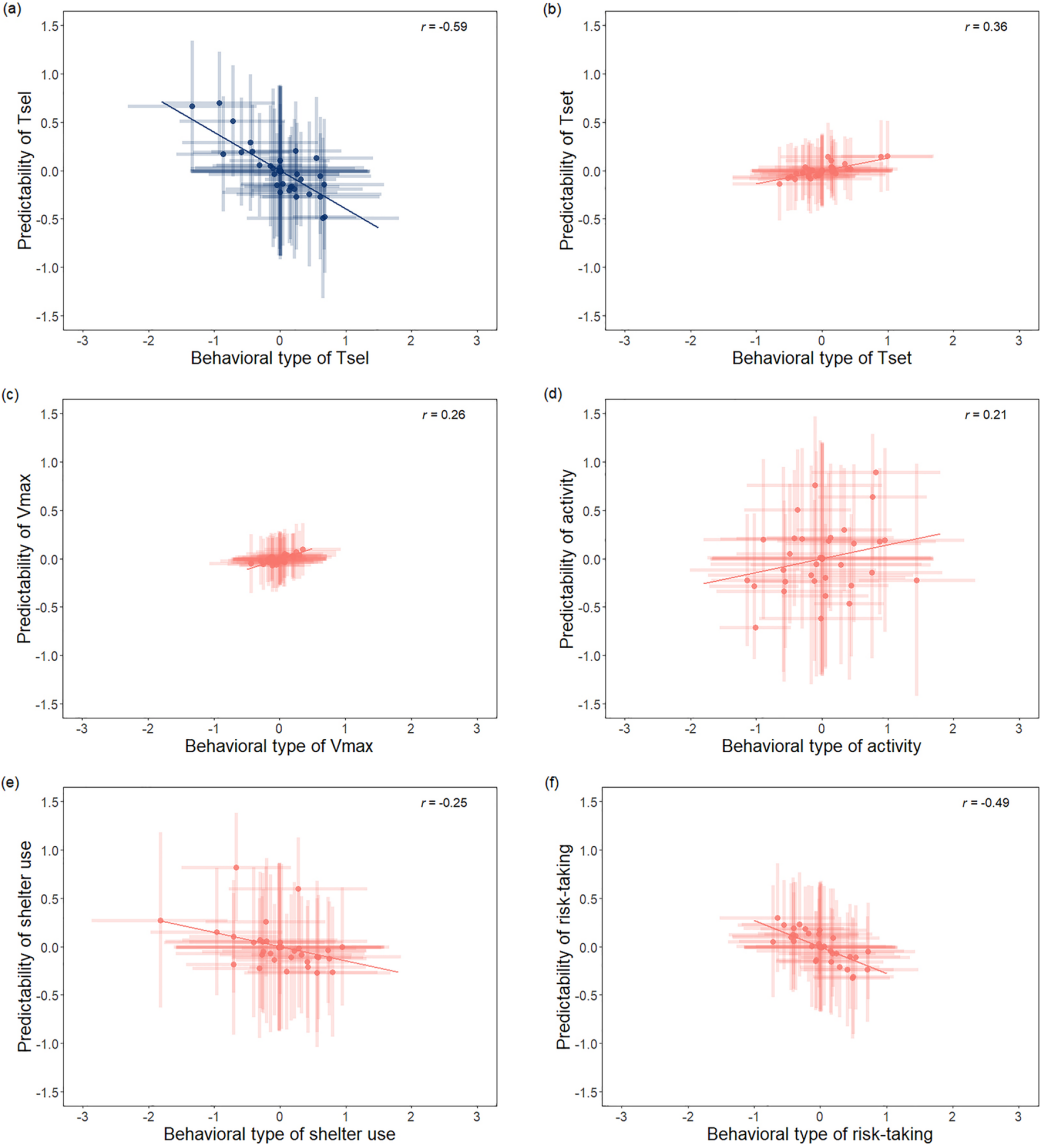
Among individual correlation (*r*) between behavioural type and behavioural predictability in (**a**) median of selected body temperatures, (**b**) set-point range (central 50% of recorded body temperatures), (**c**) voluntary thermal maximum temperatures (highest body temperature reached during the experiment), (**d**) activity, (**e**) sheltering and (**f**) risk-taking of adult male *Zootoca vivipara*. Posterior means and 95% credible intervals are shown. The sole significant correlation is highlighted in blue colour. Note that low predictability values indicate high predictability.

The predicted standard deviation from the mean residual standard deviation (rIIV) varied across individuals (ω^2^_Intercept_; see Table 1), demonstrating differences in the behavioural predictability across individuals in every studied behavioural trait. A recent meta-analysis from Mitchell et al.^24^ indicates that variation in rIIV is common, the overall predicted CV_P_ being 0.26 for behavioural traits. Our estimates indicate high variance in rIIV for selected body temperatures (CV_P.Tsel_ = 0.46 [0.2 to 0.76]): i.e., some individuals are more, while others are less predictable. Conversely, variance was rather low, but still significant, for setpoint-range and voluntary thermal maximum (CV_P.Tset_ = 0.16 [8.88 × 10^–5^ to 0.34]; CV_P.TVmax_ = 0.12 [1.21 × 10^–5^ to 0.28]. Regarding ‘classic’ behavioural traits, estimates indicate especially high variance in rIIV for movement activity (CV_P.activity_ = 0.64 [0.39 to 0.87]), while moderate variance for sheltering (CV_P.sheltering_ = 0.45 [0.22 to 0.72]) and risk-taking (CV_P.activity_ = 0.32 [0.007 to 0.59]). We did not find correlations between behavioural type and predictability except for T_sel_, where these two components were negatively correlated (− 0.59 [− 0.92 to − 0.09]; see Fig. 1). As low rIIV values indicate high predictability, this correlation translates to individuals selecting higher body temperatures being also more predictable.

### Behavioural syndromes

Regarding non-zero between-individual behavioural correlation (i.e., behavioural syndrome) across behavioural traits describing thermoregulation and behaviour, our multivariate mixed model revealed a significant T_sel_–risk-taking syndrome (r [95% credible intervals] = − 0.34 [− 0.71 to − 0.02], see (Table 2). Note that risk-taking is a latency variable, hence, the negative correlation translates to a positive link between the two traits, i.e., individuals with higher selected body temperatures were also taking more risk. A significant between-individual correlation was also present between T_sel_–T_Vmax_ (0.45 [0.07 to 0.78]), i.e., lizards with higher (median) selected body temperatures also tolerated higher maximum temperatures. Moreover, we found indication for the presence of behavioural syndromes between all ‘classic’ behavioural traits: activity–sheltering (− 0.77 [− 0.85 to − 0.45]), indicating that active individuals shelter less; activity–risk-taking (− 0.59 [− 0.76 to − 0.25]), indicating that risk-prone individuals are also moving more; and sheltering–risk-taking (0.64 [0.29 to 0.81]), indicating that risk-prone individuals shelter less. Between-individual correlations did not differ significantly from zero in the case of other behavioural trait pairs, and we did not detect any significant within-individual correlations (see Table 2).

## Discussion

Despite a growing interest in integrating thermoregulatory strategy into the animal personality framework, studies aimed at testing consistent individual differences in both between- and within-individual variation in thermoregulatory traits, or the between- and within-individual correlation between them are rather scarce. To assess behavioural predictability in various thermoregulatory traits, it is important to provide controlled conditions and to test individual thermoregulatory behaviour multiple times (i.e., ≥ 6). Here, based on a complex study involving adult male *Z. vivipara* lizards housed under standardized conditions, we demonstrate that consistent individual differences are present in traits describing thermoregulatory strategy (T_sel_, T_set_, T_Vmax_) as well as in movement activity, sheltering and risk-taking, supporting the presence of animal personality in all studied traits. Further, our study individuals also differed consistently in their behavioural predictability in all thermoregulatory and ‘classic’ behavioural traits. Individual state generally did not affect behavioural type or predictability, with one exception: individuals with high body condition showed higher sheltering predictability than their low-condition conspecifics. We found one significant positive correlation between behavioural type and predictability: individuals selecting higher body temperature were more predictable. Finally, we found behavioural syndromes within classic behavioural traits and within thermoregulatory traits; also, a positive syndrome between risk-taking and selected body temperature. Below, we discuss our results in the order of questions raised.

### Animal personality in ‘classic’ behavioural vs. thermoregulatory traits

In line with our predictions, adjusted repeatability estimates indicated the presence of consistent individual differences (i.e., animal personality) in all studied components of the thermoregulatory strategy of *Z. vivipara* males. Based on the classic meta-analysis from Bell et al.^63^, the average repeatability of behavioural traits is 0.37. Repeatability of ‘classic’ behavioural traits is estimated routinely now, however, there is a lot less data available on thermoregulatory behaviour. According to previous studies conducted under conditions and time scale similar to ours, individual differences in selected body temperature (T_sel_) were found to be present, but only weakly repeatable (R = 0 to 0.26) in four species of Cordylid lizards^64^ and in *Lampropholis delicata* (R = 0.07^32^). Contrary to these reports, our estimate (R = 0.66) can be considered high. Repeatability of thermal tolerance (T_Vmax_) to our knowledge was assessed only in a salamander, *Plethodon metcalfi*^65^, reported estimates showing low to moderate repeatability (R = 0.32). In comparison, our estimate (R = 0.87) can be considered particularly high. Thermoregulatory precision (T_set_) was also highly repeatable (R = 0.83). Previous findings based on estimates from six squamate species show that the repeatability of this trait tends to vary within a wide range (R = 0.23 to 0.66^39^). High repeatability of T_set_ had been reported from *Agama atra* (R = 0.64^39^) and *Z. vivipara* (R = 0.6^37^). Regarding ‘classic’ behavioural traits, our estimates were found to be rather high (activity: R = 0.63; sheltering: R = 0.69; risk-taking: R = 0.82), considering previous estimates in *Z. vivipara* (activity: R = 0.48 to 0.71^66^ and R = 0.56^67^; risk-taking: 0.43 to 0.58^66^), or in other species (*L. delicata*, R = 0.49^68^; *Iberolacerta cyreni*, R = 0.69^69^; R = 0.34^70^), especially in case of risk-taking (*Tiliqua rugosa*, R = 0.27–0.51^71^; *L. delicata*, R = 0.09 to 0.15^68^; *I. cyreni*, R = 0.22 and 0.36,^69,70^).

The most important question arising here is what mechanisms explain the massive individual differences in both thermoregulatory and ‘classic’ behavioural traits in our population of *Z. vivipara*. Here, we can only speculate on this without endorsing a single decisive mechanism. It has long been disputed whether consistent behavioural differences tend to emerge under challenging or under optimal environmental conditions. While available experimental results are contradictory^70,72^, it is hypothetically plausible that mild environmental stress (i.e., food shortage, presence of predatory cues, low thermal quality environment, etc.) can trigger the emergence of different behavioural strategies in the populations, increasing between-individual behavioural variation. Therefore, if the studied *Z. vivipara* population experience the above-mentioned stressors, these might potentially explain the high repeatability of the studied traits, both thermoregulatory and ‘classic’ behavioural. Anyhow, many widely distributed generalist species show specialized individual strategies^73^, which may help the population to persist, especially under harsher environmental conditions; therefore, strong individuality in thermoregulatory strategies (and other behavioural traits as well) in *Z. vivipara* might explain—besides the very effective thermoregulation (see e.g.^61^)—why this species is the most widely distributed lizard.

### The effects of individual state on behavioural type and predictability

Although *Z. vivipara* showed large heterogeneity in all six studied behavioural traits, we did not find any indication that differences in state-linked traits explain variability in behavioural types, in spite of the fact that links between traits describing the individual state and thermoregulatory strategy were shown to be widespread (e.g., parasite load^34,62,74^; body size^34,75–77^). Body size (and relative size of various body parts) have a substantial impact on the thermoregulation of reptiles. Larger species (≥ 10 kg) were found to be very effective and accurate thermoregulators, although also sensitive to overheating^10,78–80^. In smaller species, however, the association between thermoregulation and body size is rather unresolved^34,80^. One possible explanation for the absence of any effect of body size and size of different body parts on behavioural type in our case is the low variation in SVL, body weight and head-size measures across our adult study lizards.

Parasite load also has been shown to have a considerable effect on thermoregulatory strategy^34,62^ and boldness as well^69,81,82^. *Karyolysus* and *Schellackia* (family Karyolysidae, suborder Adeleorina, subclass Coccidiasina, phylum Apicomplexa) are intracellular protozoans that are parasitizing a wide array of reptiles (see e.g.^83,84^). Their prevalence is usually high in *Z. vivipara* (here 80.6%; see also^85,86^). These parasites usually cause decreasing haemoglobin concentration in the host, but do not affect survival directly. Although infection intensity (parasites per 1000 erythrocytes) showed considerable variation across the study lizards (4.75 ± 4.34, mean ± SD), and Horváth et al.^34,69^ previously found these protozoans to affect risk-taking personality in *I. cyreni* males, the average intensity of infection in *I. cyreni* was substantially higher (17.65^69^) than that we found here. Thus, the relatively low infection intensity may be simply low to affect behavioural strategies in the studied *Z. vivipara* population.

Low body condition individuals sheltered less predictably than their high body condition conspecifics. Such patterns are often explained in an antipredator context: on one hand, individuals of many species react to predators by increasing sheltering time^87,88^, but decreased behavioural predictability (high rIIV) may hold some antipredator advantage as well: as predators often rely on predictable patterns in prey activity, unpredictable animals may reduce the probability of being captured^72,89–91^ (but see^92^). In line with this notion, we recently found that low body condition *I. cyreni* juveniles express their activity less predictably^71^, most probably because they cannot flee as effectively as their peers. Thus, they compensate by expressing their behaviour in an unpredictable way. In light of our current results, some may argue that increased sheltering itself could lead to lowered body condition, as previous studies have demonstrated its negative effects on body mass^93^. Nevertheless, as we did not find direct relationship between the duration of shelter use and body condition, it is less plausible that unpredictable sheltering per se resulted in low body condition. Instead, low body condition males likely have lower escape probability and unpredictable sheltering is part of their antipredator toolkit.

### Behavioural type—behavioural predictability correlations

We found indication for the presence of significant inter-individual differences not just in behavioural type, but in predictability of all studied traits too. This suggests that lizards not only vary in their behavioural mean, but also in how predictably they express thermoregulatory strategy and ‘classic’ behavioural traits. This is in line with the accumulating empirical evidence that behavioural predictability is an important component of an individual’s behavioural strategy and is a potential subject of natural selection^94,95^. We observed a strong behavioural type—predictability correlation in thermal preference, indicating that individuals preferring lower body temperatures are also less predictable in their preferences. We found no correlation in the case of other thermoregulatory or ‘classic’ behavioural traits. In a recent phylogenetic meta-analysis, Horváth et al.^26^ showed that there is large heterogeneity in behavioural type—behavioural predictability correlations across taxa, but the lack of such links seems to be generally rare. Hence, independent evolution of behavioural type and predictability is possible in the studied *Z. vivipara* population. Nevertheless, targeted studies are needed to clarify this question. In small-sized reptiles, preference for lower body temperatures was proposed to be an antipredator strategy, because a lowered ‘goal’ for behavioural thermoregulation would allow smaller individuals to avoid basking for long periods, or at sites that are conspicuous for predators^96^. Therefore, low preferred body temperatures coupled with low predictability (i.e., high variability) of thermal preference might protect animals from their potential predators, such as birds and mammals. As far as we know, there are no other experimental studies to date that directly test thermoregulatory behavioural type—predictability correlations. Therefore, more effort is needed to test hierarchical variation in thermoregulatory behaviour in order to interpret the ecological value of such patterns.

### Behavioural syndromes within and across personality vs. thermoregulatory traits

Regarding ‘classic’ behavioural traits, we found strong behavioural syndromes in all possible combinations, implying that the studied behavioural traits cannot respond to selection independently. Previous results revealed no general existence of behavioural syndromes in *Z. vivipara*: Mell et al.^66^ found no correlation between activity, aggression, risk-taking and sociability, while Cote et al.^97^ and Le Galliard et al.^55^ report linkage between sociability and risk-taking, or activity and risk-taking (respectively). The variation in presence/absence of syndromes indicates that the emergence of behavioural syndromes results more from local adaptations than constraints in *Z. vivipara*. However, the above mentioned studies checked phenotypic correlations, while testing ‘true’ behavioural syndromes should be based on separating within- and between-individual correlations^98^. Regarding reptiles, there’s only a handful of studies that accomplish this requirement (see^70,99–101^), hence, our results provide important contribution to evolutionary ecology of behavioural syndromes in reptiles.

We also found indication of a syndrome between selected body temperature and voluntary thermal maximum (lizards with higher preferences tolerate higher maximum temperatures), but more importantly, selected body temperature and risk-taking were also positively correlated, suggesting that risk-prone lizards also preferred higher body temperatures, in line with our predictions. As mentioned earlier, it is plausible that thermal behavioural types are underpinned by a metabolic link^32,35,42^, but other experimental results show a controversial picture^37,48^. In our case, an ecological or evolutionary link is also plausible. For instance, risk-prone *Z. vivipara* males can attain higher preferred temperatures by occupying better and more conspicuous basking spots, and continue to thermoregulate even during risky situations. Their attained high body temperature translating to optimal locomotor performance would allow them to escape successfully, making the risk-prone strategy working. Although we did not find any direct link between thermoregulatory precision and any of the ‘classic’ behavioural traits, as was described before, *Z. vivipara* males who preferred higher temperatures were also less variable. Hence, it indeed seems that following a risk-prone behavioural strategy does not ‘allow’ high variability in thermoregulation, likely because of biological constraints. We note that within-individual correlations were not detected in our system, suggesting that different behaviours can vary independently from each other within individual, hence, individual behavioural plasticity is not constrained. The lack of within-individual correlations also mean that phenotypic correlations are entirely caused by relationships between individuals’ average levels of different behaviours (i.e., between-individual correlations^98^) and thus are based on stable individual behavioural strategies. This also suggest that, unlike individual behavioural plasticity, population level behavioural evolution is constrained.

### Conclusions

We found strong animal personalities in thermoregulatory strategy and ‘classic’ behavioural traits. Further, we found consistent between-individual variation in behavioural predictability in both ‘classic’ behavioural traits and thermoregulatory strategy. These findings imply that in *Z. vivipara*, or at least in the study population, individual behavioural strategies are strongly divergent. Individual state—behavioural strategy and behavioural type—behavioural predictability links were rare. The former finding suggests that individual behavioural strategies might be rather inherited than induced by the environment or the actual state of an individual, while the latter suggest that the two components of individual behavioural strategy might respond to natural selection independently. Among the classic personality traits, we found strong behavioural syndromes in all possible combinations. This implies that the studied personality traits are under evolutionary constraint, as they cannot respond to selection independently. The positive correlation between the preferred body temperature and voluntary thermal maximum suggests that there is a thermal risk-taking syndrome in our population. We also found a strong syndrome across thermoregulatory and ‘classic’ behavioural traits, suggesting that in poikilotherms, where physiological performance is dictated by body temperature, risk-taking behavioural strategy is necessarily including higher preferred body temperature, which (1) itself is attainable by taking higher risk and (2) aids to decrease the costs of the risk-taking strategy via increased physiological (e.g., locomotor) performance. Note that we could not find within-individual behavioural correlations, further strengthening our previous conclusions about the strongly divergent individual behavioural strategies. We recommend the incorporation of thermoregulatory behaviour into personality framework in actively thermoregulating poikilotherms with equal weight. We suggest that *Z. vivipara* is a promising model to study these questions in further detail. The importance of thermoregulatory behaviour is obvious under the ongoing climate change, and by applying the sophisticated framework of animal personality studies, we would gain valuable insights into this behaviour’s ontogenetic and evolutionary flexibility. Therefore, more studies like ours and the other pioneering studies^32,35,42,102^ are warranted to reach generalisations, and upon enough data, to reveal phylogenetic patterns.

## Materials and methods

### Study animals

Adult *Z. vivipara* males (N = 36) were noosed or captured by hand on the 8th of August 2018 at the Bucin Pass, Gurghiu Mountains (46.654877 N, 25.294263 E; 1250 m asl), Romania. This period is after the annual mating season. Lizards were housed individually indoors in black plastic boxes (67 cm × 35 cm × 29 cm; length × width × height, respectively) that later served as the enclosures for the tests (see below). In the boxes, we used 8–16 mm thick clay pebbles as substrate (Agro CS Keramzit), which maintains a level of moisture favourable for this species, covered with 2–3 cm of chemical-free garden soil (Agro CS Natura series).

### Individual traits

Snout-vent-length (SVL; mean ± Standard Deviation [SD]: 5.2 ± 0.63 cm) and head size (represented by pileus length [mean ± SD: 1.11 ± 0.059 cm] and width [mean ± SD: 0.64 ± 0.03 cm], and maximum head width [mean ± SD: 0.8 ± 0.057 cm]) were measured using a digital calliper to the nearest 0.01 mm. To characterize the head size, we ran a principal component analysis (PCA) on the three measures above. We got a single principal component (PC) with strong positive loadings (proportion of variation explained = 62.79%; factor loadings > 0.72), and we used this PC as our head size variable. A digital scale was used to measure body weight (BW) to the nearest 0.1 g (mean ± SD: 4.39 ± 0.67 g). Blood samples were obtained by ventral puncture of the caudal vein with disposable sterile syringes. We made smears and air-dried them until coagulation. After 60 s, we moved them into a plastic slide box and let them to dry. Smears were fixed and stained with a haematological stain set designed for determination of differential blood cell count (Reag-Quick Panoptic set, Reagens Kft). Parasite intensity was estimated by counting the number of infected red blood cells per 1000 randomly selected red blood cells under the light microscope during three scanning sessions on each smear^103^. The average of the three sessions was used to represent individual parasite infection intensity.

### Measurements of thermal behaviour

After measuring individual traits on the day of capture, lizards were released to their housing boxes on the 9th of August. We maintained the ecological costs of thermoregulation negligible by providing food (mealworms, *Tenebrio molitor* and house crickets, *Acheta domesticus*) and water ad libitum, and excluding all constraints stemming from predation and competition to get an unbiased estimate of thermal preference^9^. Acclimation was allowed during the next two days prior to the start of the measurements. Selected body temperatures were measured along a thigmothermal gradient provided in the housing boxes. To eliminate the effect of light as a potential confounding factor, heat sources were provided by infrared bulbs (Exo Terra Infrared Basking Spot 50 W, [Rolf C. Hagen Ltd., UK]; operating from 7:30 am to 7:00 pm [UTC + 02.00]). The infrared lamps created a nearly linear thermal gradient from 23 °C to an average of 55–60 °C maximum temperature, which is wider than all thermal preference ranges known for heliothermic lizards^12,13,61,104–107^. Although *Z. vivipara* possess visual sensitivity in the near infrared spectrum^108^, the maximum of the relative spectral power of our infrared bulbs is at ca. 800 nm, which is outside of the relative sensitivity of the species’ long-wavelength sensitive cones. Thus, our heating bulbs likely did not disrupt normal activity of the lizards. A natural 12 h light–dark cycle (7:00 am to 7:00 pm), was provided by a small white light (Total Green, Romania; 40 W, 300 lm) positioned in the centre of the arena. We also provided a longitudinal shelter (one per box) of 55 × 15 cm along the whole thermal gradient. The shelters could be quickly removed with minimal disturbance to the animals, thus thermal measurements were also performed under the shelter.

The thermal behaviour measurements were recorded on even days between the 12th and 22nd of August 2018, for 6 days altogether. The selected body temperatures were obtained with a non-contact infrared thermometer (Fluke® 568 Infrared Thermometer) every 30 min from 8:00 am to 6:30 pm. We hose to record the surface temperature rather than the cloacal body temperature because the latter would have required frequent handling of the lizards, potentially altering their behaviour^37^. Previous results show that body surface temperature around the midbody correlates strongly with core body temperature in small sized heliothermic lizards^12,13,109,110^.

To describe thermoregulatory behaviour, we calculated for every individual on every measurement day separately (1) the selected body temperatures (T_sel_) as the median of body temperatures, (2) the voluntary thermal maximum (T_Vmax_), defined as the highest body temperature reached and (3) the width of the set-point range (T_set_) defined as the central 50% of recorded body temperatures. We treated T_sel_ as the ‘goal’ of behavioural thermoregulation, T_Vmax_ as a proxy for an individual’s thermal tolerance and T_set_ as a measure of thermoregulatory precision.

### Behavioural assays

Sheltering, movement activity and risk-taking behaviour of lizards were assayed on odd days between the 13th and 21st of August for five days altogether. We assessed behavioural traits based on 60 min videos that were recorded between 10:00 am and 1:00 pm (activity), and 1:00 pm and 4:00 pm (risk-taking) using camcorders (Panasonic HC-V160, Panasonic Co.). We were able to record 12 lizards at a time, and the groups were chosen randomly during the 3-h trial series. We assessed general activity by evaluating the time (sec) the animals spent under the shelter during a 60 min recording period (thereafter ‘sheltering’). Sheltering was estimated as the sum of the time spent hiding (body and head inside the refuge). To assess activity, the ground area of the housing boxes was divided into 9 equal virtual grid squares and the number of transitions between squares was counted^32^. We used the formula ‘(number of transitions/time (sec) spent outside) × 60’ to get a measure of movement activity independent from sheltering. Risk-taking behaviour was estimated by exposing lizards to a simulated predatory attack (the experimenter caught the lizard by hand and put it under the refuge). Time (sec) till the lizards left their shelters and started basking was used as a proxy of risk-taking with individuals emerging and start basking quickly seen as risk takers. The recording was processed and analysed in *MWrap* software^111^.

### Statistical analysis

All thermal (T_sel_, T_Vmax_, T_set_) and ‘classic’ behavioural traits (activity, sheltering and risk-taking) were estimated separately for each assay day for each individual to obtain repeated estimates. Sheltering and risk-taking were log-transformed to achieve a normal distribution of the model residuals.

Repeatability estimates, state variable effects and correlations between behavioural type and predictability were estimated by double hierarchical mixed models (DHMMs). We ran separate DHMMs on our thermoregulatory and ‘classic’ behavioural traits using the R package *brms*^112,113^ based on the Bayesian software Stan (version 2.26^114^). In this approach, the ‘mean model’ estimates whether individuals differ in their mean expression of behaviour (i.e., behavioural type), while the ‘residual model’ estimates whether they differ in residual intra-individual variation (rIIV) around this behavioural mean (i.e., behavioural predictability; for more details on DHMMs see^92,93,115^). Further, we estimated the correlation between the random intercepts in the mean model and residual model (i.e., the correlation between behavioural mean and rIIV; e.g.^26^). Traits describing thermoregulatory strategy, activity, sheltering and risk-taking were used as response variables in the models and analysed using Gaussian distribution. Both response- and continuous explanatory variables were standardized (i.e., z-transformed, mean = 0 and standard deviation = 1). This transformation does not affect the parameters of interest but aids model fitting. To assess the potential effect of state-linked individual traits on behavioural type and behavioural predictability, both mean and residual level models were parametrized similarly. We added a population intercept (mean model: *β*_*0*_, residual model: *γ*_*0*_), with the fixed effects SVL, body condition (relative BW; estimated as regression residuals from BW−SVL regression), relative head size (estimated as head size−SVL regression), mean blood-parasite load, allowing two-way interactions between the blood-parasite load and the individual size measures. Time (i.e., test day) was fitted to the mean level models to test for potential habituation effects as well (while not to the residual models). Further, we tested individual differences in habituation by including time as a random slope (i.e., in interaction with individual identity) to the mean models. In the residual models, the hyperparameter *ω*^*2*^ reflects on the log scale, how strongly individuals differ in their residual standard deviation, measured on the log scale, i.e., it allows for assessment of the consistency of individual differences in predictability through time^116^.

Adjusted repeatability estimates of traits describing thermoregulatory strategy and behaviour were calculated from the DHMMs by dividing variance explained by among individual level by the total phenotypic variance^117^.

$$R = V_{individual} /\left( {V_{individual} + V_{residual} } \right)$$

Note that the residual variance (V_residual_) here refers to the population intercept of the residual model (γ_0_), hence this intercept was converted into a variance by taking its exponent and squaring the resulting value since the dispersion model uses a log scale to estimate residual standard deviations.

We estimated individual variation in predictability as the coefficient of predictability (CV_P_), which is a standardised metric quantifying among-individual variation in the predicted standard deviation from the mean rIIV.

$$CV_{p} = \sqrt {\left( {exp\left( {\omega^{2} } \right) - 1} \right)}$$

The posterior distribution of the rIIV for each level of the random intercept as an indicator for behavioural predictability was extracted. When rIIV is high, the residual standard deviation around the behavioural mean is high indicating higher behavioural variability (i.e., low behavioural predictability).

For the DHMMs, we used weakly informative normal priors (N (0,1)) for fixed effects, half-normal priors (N (0,1)) for random effects, and a Lewandowski-Kurowicka-Joe (LKJ(1))−correlation prior for the correlation of random effects^118^. We ran four chains to evaluate convergence, which was run for 5000 iterations, with a warmup of 1000 iterations and a thinning interval of 4. All estimated model coefficients and credible intervals were therefore based on 4000 posterior samples and had satisfactory convergence diagnostics with R-hat (potential scale-reduction factor on split chains) < 1.01, and effective sample sizes > 400^119^. We report the mean and 95% credible intervals, calculated as the highest posterior density intervals, for all parameters in our statistical models to assess whether the parameters were statistically different from 0.

To test for among-trait (co)variation at the between- and within-individual level, we ran a multivariate mixed-effect model. In this model, the six behavioural traits of interest were fitted as response variables, while the individual was added as a random effect, using the *MCMCglmm* function from the *MCMCglmm* R package^120^, which implements a Bayesian framework for model fitting with long iterations (1.300.000 with 300.000 burn-in periods); the Markov chain was sampled at each 1000th iteration. Based on our model, we decomposed phenotypic correlations into among- and within-individual correlations, using the former as an indicator of behavioural syndromes^97,121,122^. The results are given as correlation coefficients and their 95% credible intervals. Analyses were performed using R 4.3.0^123^ and IBM SPSS Statistics 29.0 (SPSS Inc., Chicago, IL).

### Statement

The study is reported in accordance with ARRIVE guidelines https://arriveguidelines.org). All applicable international, national, and institutional guidelines for the care and use of animals were followed. The experimental protocol was approved by the ethics committee of the “Milvus Group” Bird and Nature Protection Association (permit number: 237/2018). No lizard was injured during the handling related to the study and after completion of the experiments, animals were returned in good health at the exact site of capture.

### Supplementary Information

Supplementary Figure S1.

## Data Availability

Data and detailed code for the analyses can be found at the Open Science Framework (OSF): https://osf.io/7pfjz/?view_only=c25b8ec86b5349749f7e9e3d0c7ba563.
