# Supplementary material for: Integrating behavioural thermoregulatory strategy into the animal personality framework using the common lizard, Zootoca vivipara as a model
Source: Sci Rep. 2024 Jun 20;14:14200. doi: 10.1038/s41598-024-64305-z (PMC11189939; doi:10.1038/s41598-024-64305-z)

Figure S1. Individual differences in a) median of selected body temperatures, b) set-point range (central 50% of recorded body temperatures), c) voluntary thermal maximum (highest body temperature reached during the experiment), d) activity, e) sheltering and f) risk-taking of adult male *Zootoca vivipara*. Boxes indicate the interquartile range (IQR), with the central line depicting the median and the whiskers extending to 1.5 * IQR.


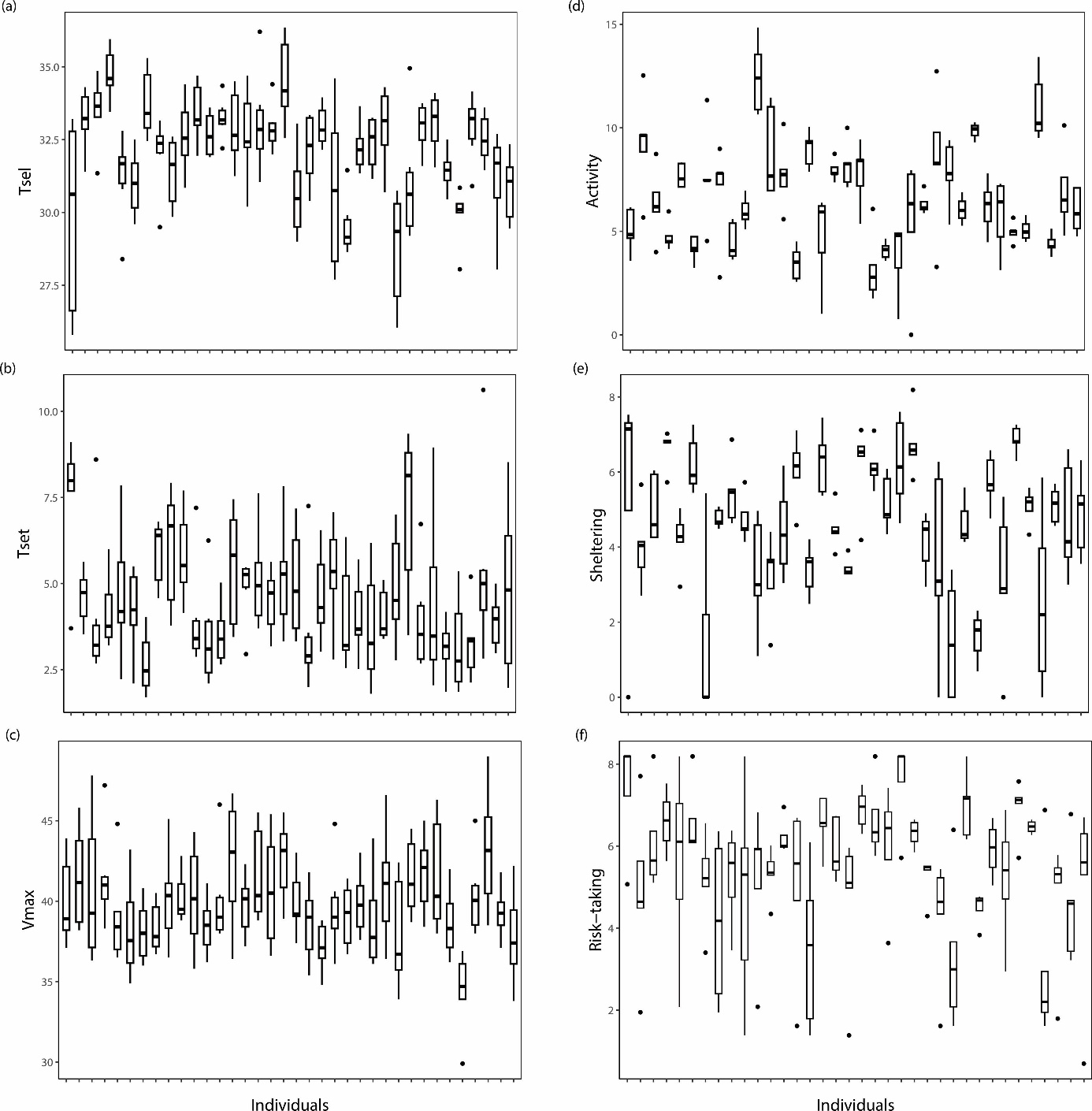

Supplement: Supplementary file 1 — Supplementary Figure S1. [file 41598_2024_64305_MOESM1_ESM.docx]
